# Microalgae as a sustainable alternative to palm oil: fatty acid profiles under photoautotrophic and heterotrophic growth

**DOI:** 10.1007/s00253-025-13682-0

**Published:** 2026-01-12

**Authors:** Karolína Štěrbová, Kateřina Bišová, Jiří Masojídek

**Affiliations:** 1 Laboratory of Algal Biotechnology, Institute of Microbiology of the Czech Academy of Sciences, Centre ALGATECH, Třeboň, Czech Republic; 2 Laboratory of Cell Cycles, Institute of Microbiology of the Czech Academy of Sciences, Centre ALGATECH, Třeboň, Czech Republic; 3 Faculty of Science, University of South Bohemia, České Budějovice, Czech Republic

**Keywords:** Microalga, Biomass, Photoautotrophic and heterotrophic cultivation, Fatty acid, Palm oil

## Abstract

**Abstract:**

Palm oil is the world’s most widely used vegetable oil, with a sizeable impact on the environment. As an alternative, microalgae are considered oil producers since they produce a variety of fatty acids (FA) depending on growth conditions. A collection of ten microalgae strains naturally producing oils similar in composition to palm oil was selected, and the effects of cultivation regime and varying light intensity on their growth and FA production and composition were analysed. To achieve high biomass density as well as total fatty acid (TFA) content, the optimum irradiance of 400 µmol photons m^−2^ s^−1^ in a photoautotrophic regime was determined for most of the strains. The growth rates of *Scenedesmus* and *Desmodesmus* strains in general were approximately twice as high as *Chlamydomonas*. The highest TFA content was found in *S. obliquus* CCALA 455 and *D. subspicatus* CCALA 467, grown photoautotrophically, reaching the values of about 66% and 58% of their dry weight, respectively. Moreover, the content of palmitic (PA), oleic (OA) and linoleic acid (LA) of about 39%, 30% and 14% of TFA, respectively, determined in *D. subspicatus* CCALA 467 was closest to that in palm oil (44% of PA, 39% of OA and 10% of LA). Eight of the ten microalgae strains were capable of heterotrophic growth, although their production under this regime has not been considered suitable in terms of TFA and individual FA content.

**Key points:**

• *The optimum irradiance of 400 µmol photons m*^−2^ *s*^−1 ^*was determined*

• *CCALA 467 produces selected FAs in amounts close to those in palm oil*

• *TFA content (% of dry weight) in CCALA 467 is 1.6-fold higher than in the palm*

**Graphical Abstract:**

**Figure:**
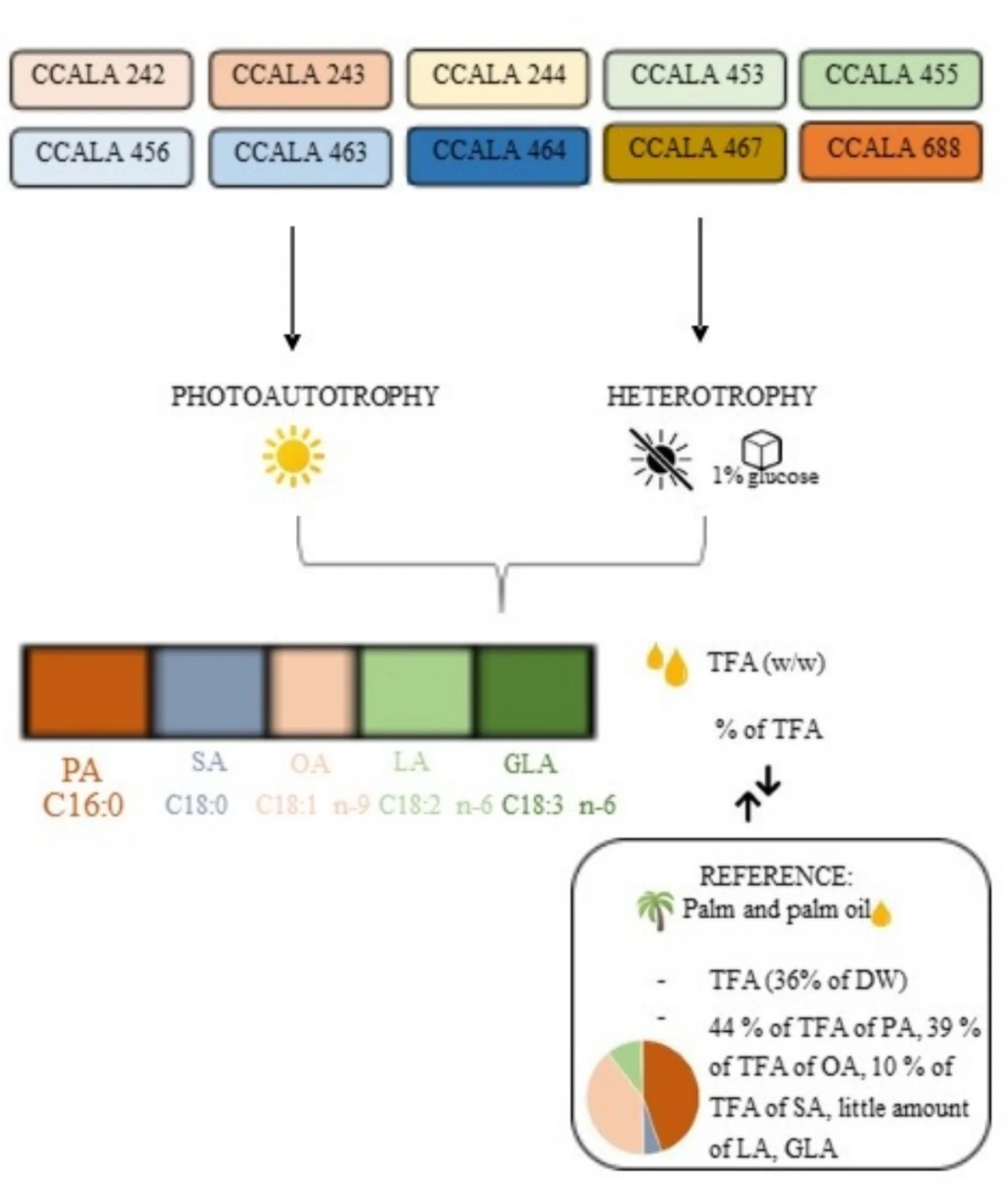

**Supplementary Information:**

The online version contains supplementary material available at 10.1007/s00253-025-13682-0.

## Introduction

Palm oil is the most widely used vegetable oil worldwide, accounting for 37% of total annual consumption with a large range of industrial applications, while oil production from soybean, rapeseed and sunflower makes up 28, 12 and 10%, respectively (Chiriacò et al. 2024). About half of all consumer products sold in supermarkets are made of palm oil (Emily 2022). The world’s leading producers of palm oil are Indonesia and Malaysia, which cover more than 85% of the global palm oil supply (Absalome et al. 2020; Sulaiman et al. 2022). By 2050, the overall demand for palm oil is predicted to reach 240 million metric tonnes (Sehgal and Sharma 2021).

Despite being inexpensive, the production of palm oil is known for its significant environmental impact. Deforestation, followed by the planting of palm trees for oil production, is a serious global issue for environmental protection. Thus, there is a serious need to search for alternative sources (Afriyanti et al. 2016; Waghmare et al. 2018). The principal constituent of palm oil is palmitic acid (PA), accounting for almost half of the amount (44%). Then, it contains oleic acid (OA; 39%), linoleic acid (LA; 10%) and the rest consists of stearic, myristic, linolenic (e.g. α-linolenic acid; ALA and γ-linolenic acid; GLA), lauric and arachidic acids (Mancini et al. 2015).

Some microalgae produce a variety of saturated (SFA), monounsaturated (MUFA) and polyunsaturated fatty acids (PUFA), and the extracted oil has a similar chemical composition to palm oil, making them an alternative source. Moreover, their cultivation is environmentally sustainable, taking up less space to grow. This makes microalgae a promising alternative source to replace or at least reduce the demand for palm oil production (Waghmare et al. 2018). As the lipid production and FA profile are related to the cultivation conditions, there is a need to find suitable cultivation conditions for individual microalgae species producing an adequate amount of targeted FA. They are vital constituents of microalgae biomass and typically account for up to 60% of cell dry weight (DW). The compounds and their amounts are species-specific (Morales et al. 2021). Some studies revealed the health potentials associated with the monounsaturated fatty acids (MUFA) from microalgae (Liu et al. 2022). PA, the most abundant FA in palm oil, can be found in several phyla such as Chlorophyta, Rhodophyta, Haptophyta, Cryptophyta, Dinophyta and Bacillariophyta if grown under nutrient-replete conditions (Bellou et al. 2014). In addition, they contain high amounts of GLA reaching values similar to or higher than PA (Ronda et al. 2012). As mentioned above, cultivation variables influence the FA profile and hence, the quality and quantity of lipids produced (Breuer et al. 2013). As we deal with photoautotrophs, irradiance is one of the most important variables for microalgae growth (Izadpanah et al. 2018; Maltsev et al. 2021), and the suitable level is species-specific, varying between tens and hundreds of micromol photons per m^−2^ s^−1^ (Morales et al. 2021; Maltsev et al. 2021). Higher light intensity mostly increases lipid content (Jiang et al. 2011; Mulgund 2022) and, together with exposure duration, is related to variations of the contents SFA, MUFA and PUFA (Amini Khoeyi et al. 2012; Morales et al. 2021).

In most cases, microalgae are cultivated in photoautotrophic conditions, but several species can also grow heterotrophically in the dark using organic carbon and energy sources, e.g. glucose, fructose, sucrose, lactose, galactose and acetic acid (Velu et al. 2015; Chen and Jiang 2017; Gao et al. 2023). The most commonly used organic substrate is glucose (Ren et al. 2013; Gao et al. 2023).

In this study, several Chlorophyta species were grown in photoautotrophic as well as heterotrophic cultivation regimes at optimum temperature in various laboratory bioreactors and the profiles of individual FA were compared with the profile of palm oil.

## Materials and methods

### Organisms and culture maintenance

Ten microalgae strains from the Culture Collection of Autotrophic Organisms (CCALA), Třeboň, Czech Republic, were used as standard cultures, belonging to the genera *Chlamydomonas*, *Scenedesmus* and *Desmodesmus* (class Chlorophyceae). They were selected based on the highest amounts of PA and OA, which are the two most abundant FAs in palm oil (Lang et al. 2011). Three strains of microalga *Chlamydomonas moewusii* CCALA 242, CCALA 243 and CCALA 244 (further abbreviated as *C. moewusii* CCALA 242, 243 and 244), three strains of *Scenedesmus obliquus* CCALA 453, CCALA 455 and CCALA 456 (further as *S. obliquus* CCALA 453, 455 and 456), two strains of *Desmodesmus communis* CCALA 463 and CCALA 464 (further as *D. communis* CCALA 463 and 464) and two strains of *Desmodesmus subspicatus* CCALA 467 and CCALA 688 (further as *D. subspicatus* CCALA 467 and CCALA 688) were studied.

In the case of photoautotrophic cultivation, the cultures were initially grown in the BG-11 medium (Hughes et al. 1958; Allen and Stanier 1968) in 250-mL Erlenmeyer flasks placed on the laboratory shaker at 22–25 °C and illuminated by continuous light of about 50 µmol photons m^−2^ s^−1^.

For heterotrophic cultivation, the strains were maintained on agar-solidified ½ ŠS medium (Hlavová et al. 2016) by subculturing every 3 weeks. The freshly streaked cultures were grown on a light shelf at an incident light intensity of 100 µmol photons m^−2^ s^−1^ photosynthetically active radiation at 22–25 °C for about a week and then stored in the dark at 15 °C before the trial.

### Photoautotrophic cultivation

In Trial 1, the light optimisation of all microalgae was performed in 100-mL glass columns with a light path of 25 mm. Each column was inoculated to the initial optical density of about OD_750_ = 0.2. The columns were submerged in a temperature-controlled water bath set to the growth optimum of 30 °C, which is common for green microalgae (Ranglová et al. 2019). The columns were mixed by bubbling air + 1% CO_2_ (v/v) with a flow rate of 50 mL min^−1^ and exposed to four light intensities—50, 100, 200 and 400 µmol photons m^−2^ s^−1^ to find the suitable irradiance. The experiment lasted for 10 days. The suitable light intensity, the total content of FAs and the content of individual FAs were determined. All cultivation trials were carried out in triplicate.

### Heterotrophic cultivation

In Trial 2, the cultures were inoculated directly from the plates into 300 mL of ½ ŠS medium and grown photoautotrophically in vertical glass columns (inner diameter = 36 mm, height = 500 mm, volume of suspension = 300 mL) at continuous light of incident light intensity of 500 µmol photons m^−2^ s^−1^, at 30 °C and were aerated with air + 2% CO_2_ (v/v). The cultures were grown until the optical density (OD_750_) reached about 0.3; then they were diluted to approximately 10^6^ cells mL^−1^ by the ½ ŠS medium containing 1% of glucose as a carbon source and placed into an RTS-8 multi-channel bioreactor (Biosan, Latvia) with the following settings: culture volume of 40 mL, temperature of 30 °C and agitation 2000 rpm. The growth of the culture was monitored as changes in optical density at 660 nm for about 90 h; however, the data is only shown for about 72 h when the stationary phase was reached in most of the species and the growth stopped. The composition of the biomass was analysed at the end of the cultivation. All cultivation trials were carried out in triplicate.

### Analytical measurements

#### Biomass density

The biomass density of the culture was determined as dry weight (DW) and the measurement was performed by filtering culture samples on pre-weighed glass microfiber filters (GC-50) as described previously (Ranglová et al. 2019). In brief, the volume of 5 mL of culture was used for DW determination. The pre-weighed filters with the cells were then washed twice with deionized water, dried in an oven at 105 °C for 8 h, transferred to a desiccator to equilibrate to laboratory temperature and weighed. The specific growth rate *µ* = (ln DW_2_ − ln DW_1_)/*t*_2_ − *t*_1_) (day^−1^) was calculated in the exponential phase of growth.

#### Analysis of fatty acids

The identification and quantification of FAs were performed in the biomass samples taken at the end of the trial. The separation of methyl esters of individual fatty acids (FAMEs) was performed on a Thermo Trace 1300 gas chromatography system as described previously (Lakatos et al. 2023). The amount of 5–10 mg of lyophilized biomass was mixed with 400 µL of zirconium/silicon beads in a breaking vial and 1 mL mixture of 3 M hydrochloric acid in methanol followed by 50 µg of internal standard (C15:0) was added. After the disintegration of microalgae cells (5 cycles, each lasting 30 s on Mini-Beadbeater-16, BioSpec Products, USA), the samples were cooled down on the ice and the content of the vial was washed twice with 1 mL of methanol. The reaction mixture was heated at 90 °C for 1.5 h in a thermoblock, then cooled to laboratory temperature and 2 mL of hexane and 2 mL of 1 M NaCl were added. After a short mixing, the sample was centrifuged at 900×*g* at 4 °C for 10 min (Eppendorf centrifuge 5804 R). The upper organic phase was separated and analysed on a TR-FAME column (60 m × 0.32 mm, df 0.25 µm) while helium was used as a carrier gas at a pressure of 200 kPa. The retention times of FAMEs were compared to known standards from menhaden fish oil (Supelco® 37 Component FAME Mix; PUFA No. 3 Supelco) and the amounts of individual FAs were calculated by multiplying the integrated peak areas by the correction factors of the FID response. All individual FAs detected in the microalgae sample at each light intensity (Table S1) as well as at heterotrophic condition (Table S2) were measured, but only the FAs identified in palm oil (detected in microalgae in amounts greater than 1% of TFA - palmitic acid - PA, C16:0; stearic acid - SA, C18:0; oleic acid - OA, C18:1 n-9; linoleic acid - LA, C18:2 n-6; γ-linolenic acid - GLA, C18:3 n-6) were considered in this study.

### Statistical analysis

All measurements were performed in triplicate (*n* = 3); the means and standard deviations (± SD) are reported in the figures. Sigma Plot 11.0 software was used to determine significant differences between treatments. Statistical analysis was performed using One-Way ANOVA and all pairwise multiple comparison procedures while using the Holm-Sidak test to assess differences between groups and interactions between variables. *P* values less than 0.05 were considered statistically significant. In graphs, the mean values designated by the same letter did not differ from each other.

## Results

In the first series of experiments (Trial 1), the cultures were illuminated by various light intensities (50, 100, 20 and 400 µmol photons m^−2^ s^−1^) to determine the suitable light intensity for individual microalgae strains in terms of biomass as well as individual FA production corresponding to the composition of palm oil. To decide if the ten pre-selected microalgae strains can grow heterotrophically, they were all cultured in a medium containing glucose (Trial 2). In all strains, except for *C. moewusii* CCALA 243 and CCALA 242, photo- and heterotrophic growth can be compared.

### Trial 1: Determination of suitable light intensity

**Fig. 1:**
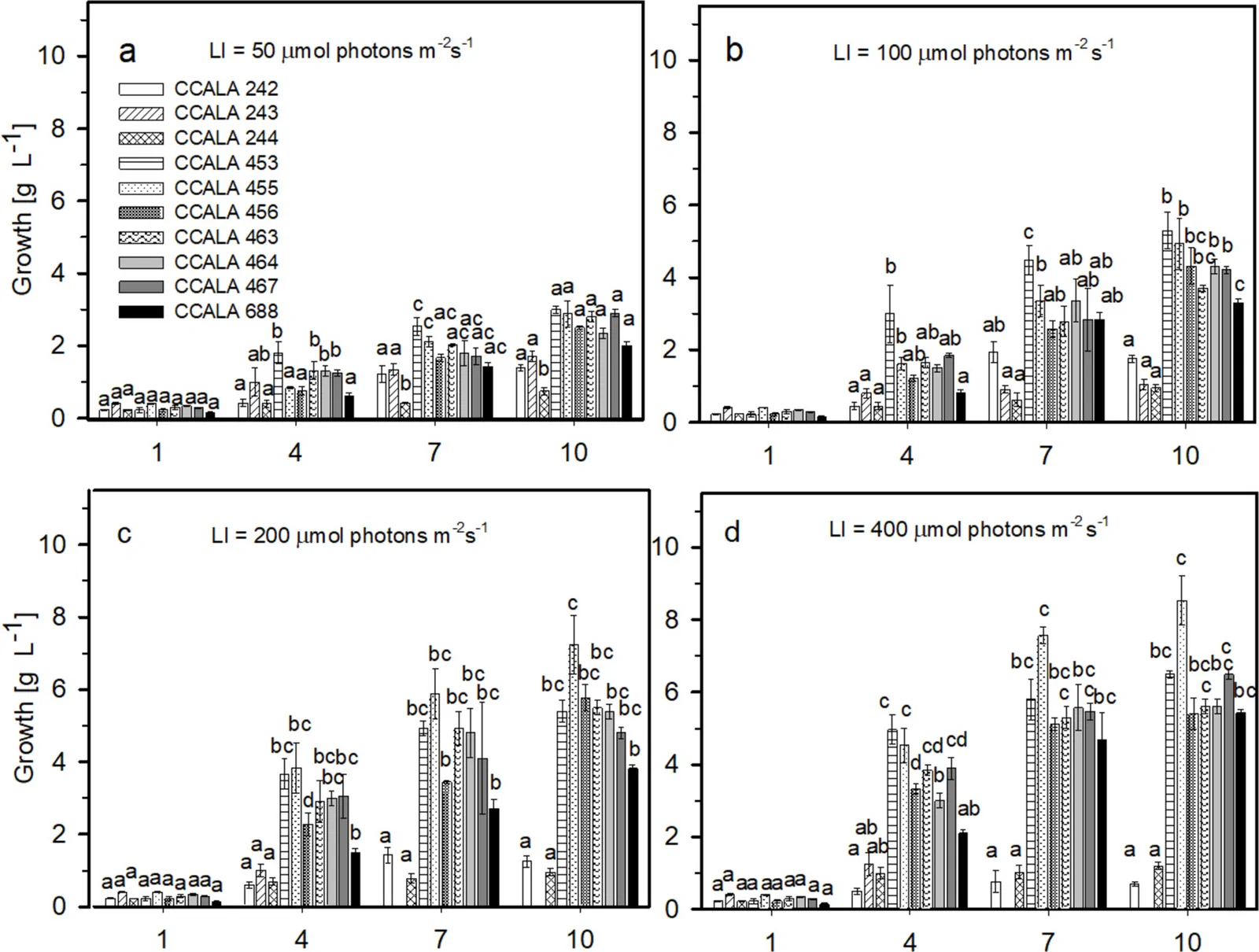
Growth of selected microalgae (*Chlamydomonas moewusii* strains CCALA 242, CCALA 243 and CCALA 244, *Scenedesmus obliquus* strains CCALA 453, CCALA 455 and CCALA 456, *Desmodesmus communis* strains CCALA 463 and CCALA 464, *Desmodesmus subspicatus* CCALA 467, *Desmodesmus subspicatus* CCALA 688) analysed at various light intensities for 10 days. Statistical analysis was performed for each day individually for the given cultivation condition. The values are presented as a mean (*n* = 3) ± SD and those designated by the same letter did not differ from each other

In Trial 1, the suitable light intensity for all microalgae was determined based on the biomass accumulation (as well as volumetric and areal productivity – Table S3). All cultures grew well except for *Chlamydomonas* species (CCALA 242, CCALA 243 and CCALA 244) (Fig. 1), as considerable sedimentation of the cells was observed (mostly in *C. moewusii* CCALA 243). In most cases, the value of 400 μmol photons m^−2^ s^−1^ was determined as suitableexcept for *S. obliquus* CCALA 456, for which the highest biomass density was measured at 200 μmol photons·m^−2^ s^−1^ (despite the highest growth rate at 400 μmol photons·m^−2^ s^−1^) and two *C. moewusii* strains (CCALA 242 and CCALA 243) in which the highest biomass accumulation was observed at 100 and 50 μmol photons·m^−2^ s^−1^, respectively.

**Fig. 2:**
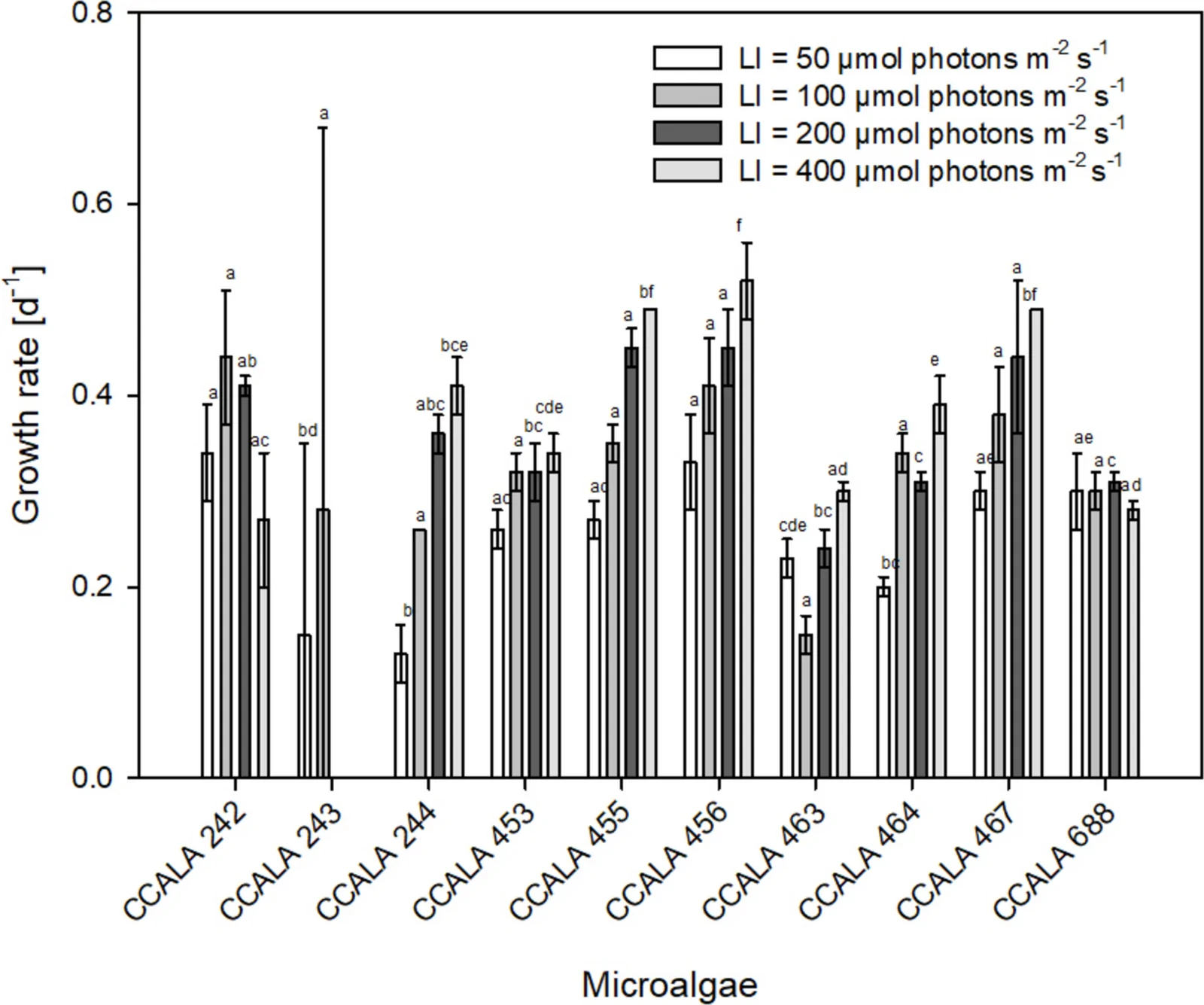
Values of the specific growth rate [µ; day^−1^] of selected microalgae (see the list in the legend of Fig. 1) when exposed to various irradiance levels of 50, 100, 200 and 400 μmol photons m^−2^ s^−1^. The values are presented as a mean ± SD (*n* = 3); those designated by the same letter did not differ from each other

The highest growth rate was determined for *S. obliquus* CCALA 456, reaching the value of µ = 0.52 ± 0.04 day^−1^ (Fig. 2) at the highest light intensity, although the highest biomass density of 5.78 ± 0.37 g L^−1^ was reached at 200 µmol photons m^−2^ s^−1^. The highest biomass density of 8.53 ± 0.68 g L^−1^ was reached in *S.obliquus* CCALA 455 (Fig. 1d) at 400 μmol photons m^−2^ s^−1^ which also corresponded to the highest growth rate µ = 0.49 ± 0.00 day^−1^ for this strain (Fig. 2). In general, the cultures of the *C. moewusii* strains did not grow well as the maximum biomass of 1.75 ± 0.10 g L^−1^ was reached in the CCALA 242 strain and the ability to grow was observed at lower radiation levels such as 50 and 100 μmol photons·m^−2^ s^−1^.

**Fig. 3:**
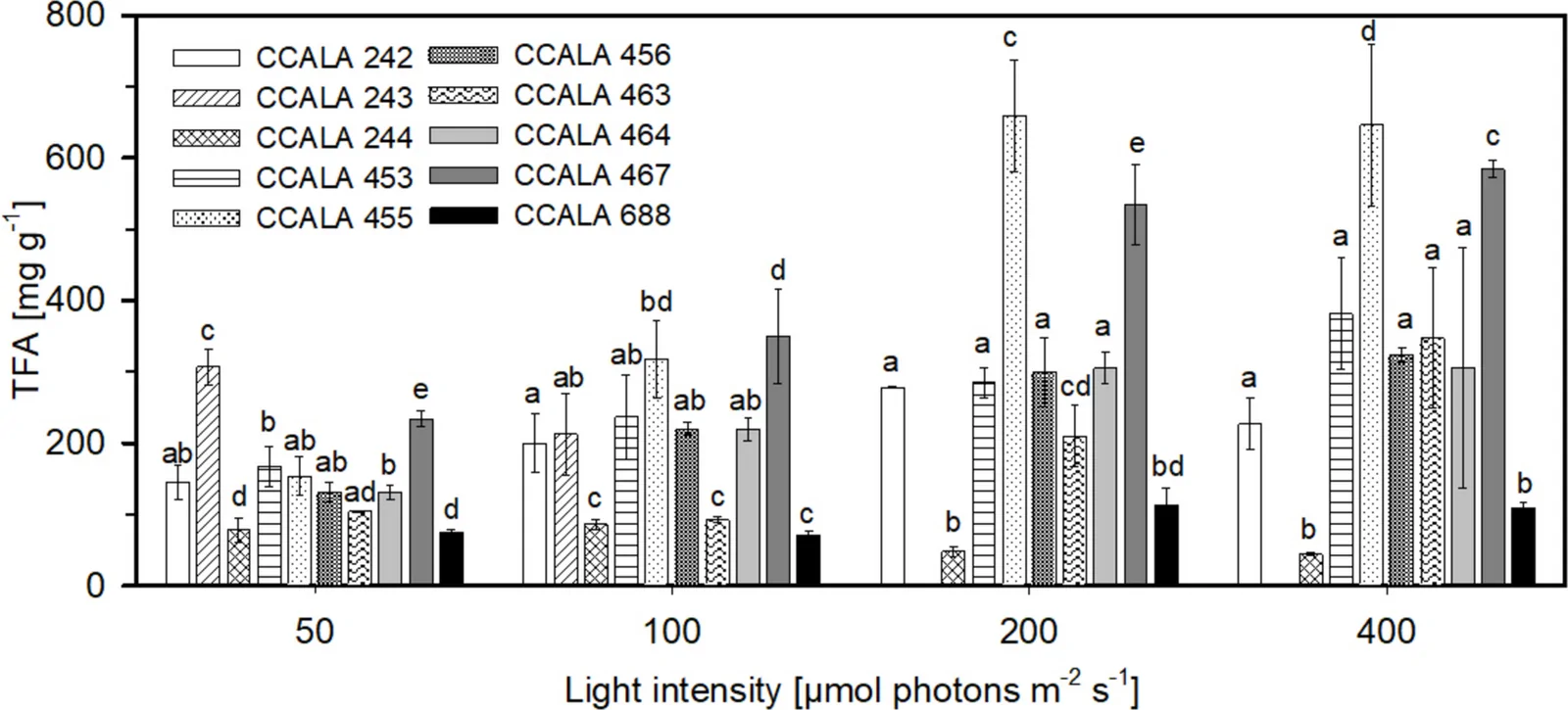
The TFA content determined in the biomass of selected microalgae (see legend of Fig. 1) at the end of the trial. Statistical analysis was performed between individual microalgae species at a given irradiance. The values are presented as a mean ± SD (*n* = 3); those designated by the same letter did not differ from each other

**Fig. 4:**
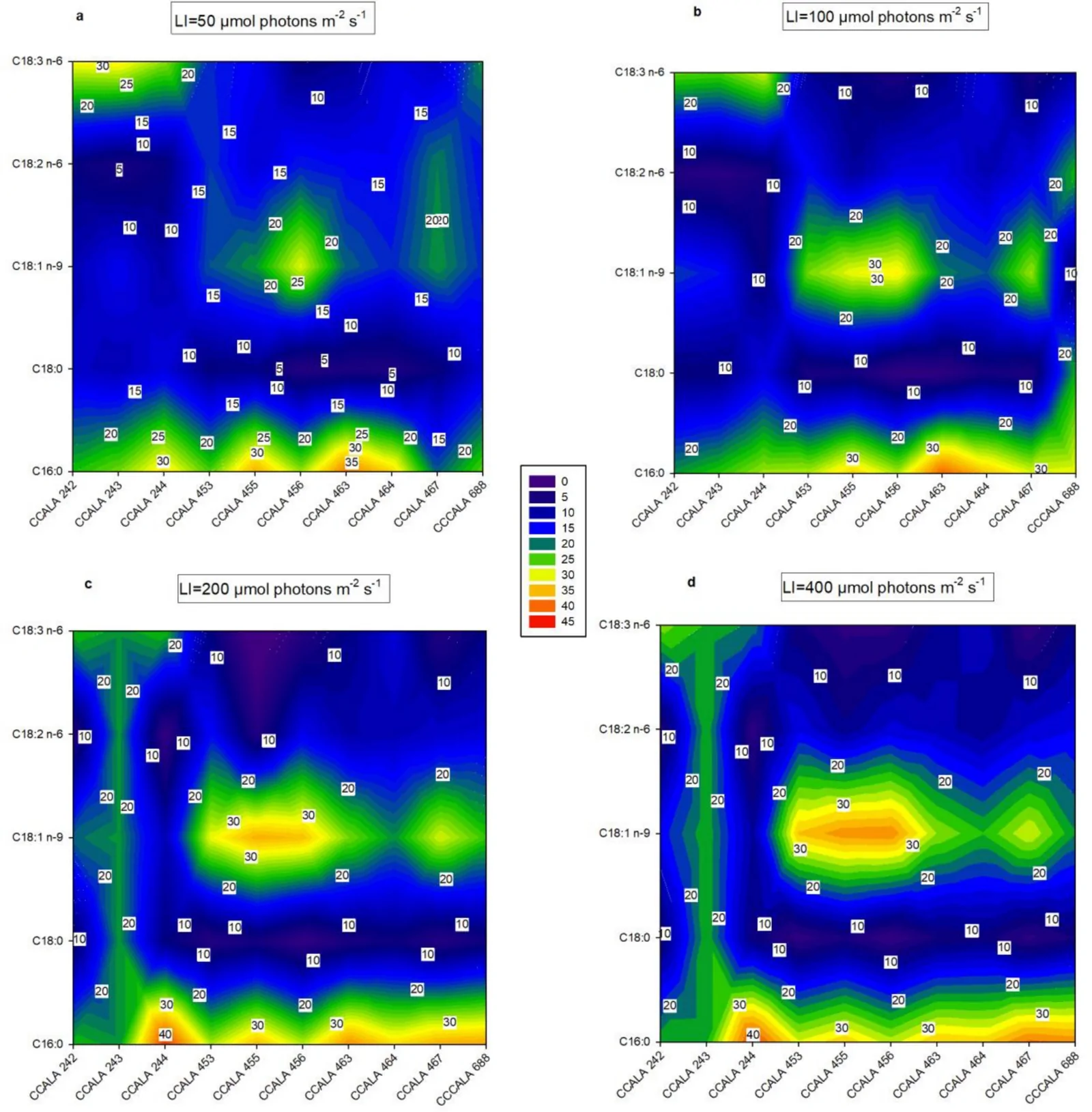
The concentration [% of TFA] of five selected fatty acids (palmitic PA C16:0, stearic SA C18:0, oleic OA C18:1 n-9, linoleic LA C18:2 n-6 and γ-linolenic acid GLA C18:3 n-6) determined in the biomass samples of the selected microalgae strains (list in the legend of Fig. 1) harvested at the end of the trial. The cultures were grown at various light intensities: **a** 50 µmol photons m^−2^ s^−1^, **b** 100 µmol photons m^−2^ s^−1^, **c** 200 µmol photons m^−2^ s^−1^ and **d** 400 µmol photons m^−2^ s^−1^

In most cases, the greatest amounts of TFA were found in the biomass cultured at optimum light intensity (Fig. 3), reaching values up to 646.2 ± 113.7 mg g^−1^ and 584.4 ± 14.0 mg g^−1^ in *S. obliquus* 455 and *D. subspicatus* 467, respectively. All microalgae strains studied here were rich in PA (Fig. 4), as the amounts found in biomass were in the range between 18.5 and 43.2% of TFA. The light intensities of 200 and 400 µmol photons m^−2^ s^−1^ favoured the production of PA. The most significant difference between *Chlamydomonas* species is observed in strain CCALA 244, where the amount of PA increased from 31.9 ± 1.9% of TFA (50 µmol photons m^−2^ s^−1^) to 43.2 ± 4.4% of TFA (200 µmol photons m^−2^ s^−1^). Of the remaining microalgae, the most significant difference was observed in *D. subspicatus* CCALA 467, where the amount of PA increased from 20.5 ± 1.6% of TFA (50 µmol photons m^−2^ s^−1^) to 37.6 ± 1.0% of TFA (400 µmol photons m^−2^ s^−1^). In *D. communis* CCALA 463, the concentration of PA did not decrease below 34% of TFA regardless of light intensity. The second most abundant FA determined in the biomass was OA, present in the range of approximately 10 to 38% of TFA. The production of OA was induced by higher light intensity, as the highest amount of 37.7 ± 1.1% of TFA was found in the biomass of *S. obliquus* CCALA 456 cultivated at 400 µmol photons m^−2^ s^−1^. The compound SA C18:0 was found in the biomass at the lowest concentration present in the range of approximately 10 to 15% of TFA.

### Trial 2: Heterotrophic trials

**Fig. 5:**
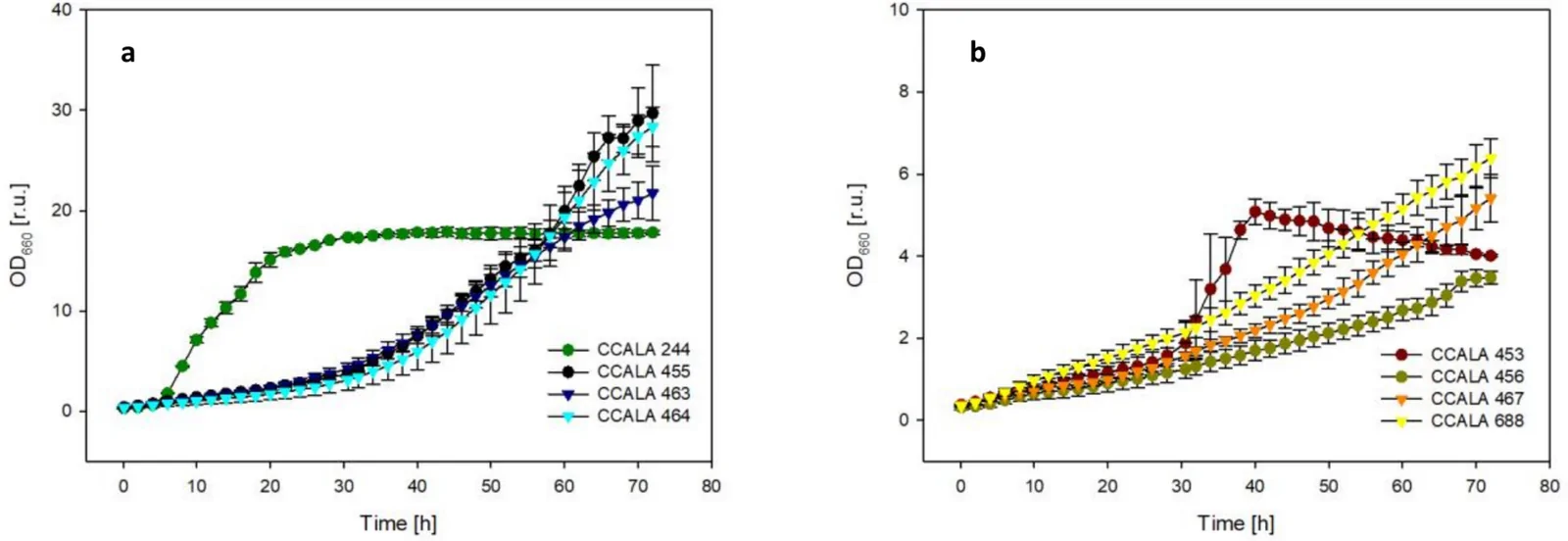
Growth of selected microalgae strains: **a** *C. moewusii* strain CCALA 244, *S. obliquus* strains CCALA 455 and *D. communis* strains CCALA 463 and CCALA 464; **b** *S. obliquus* CCALA 453 and CCALA 456, *D. subspicatus* strain CCALA 467, *D. subspicatus* strain CCALA 688—was measured during heterotrophic growth in the presence of 1% glucose for about 72 h. The values are presented as a mean ± SD (*n* = 3)

**Fig. 6:**
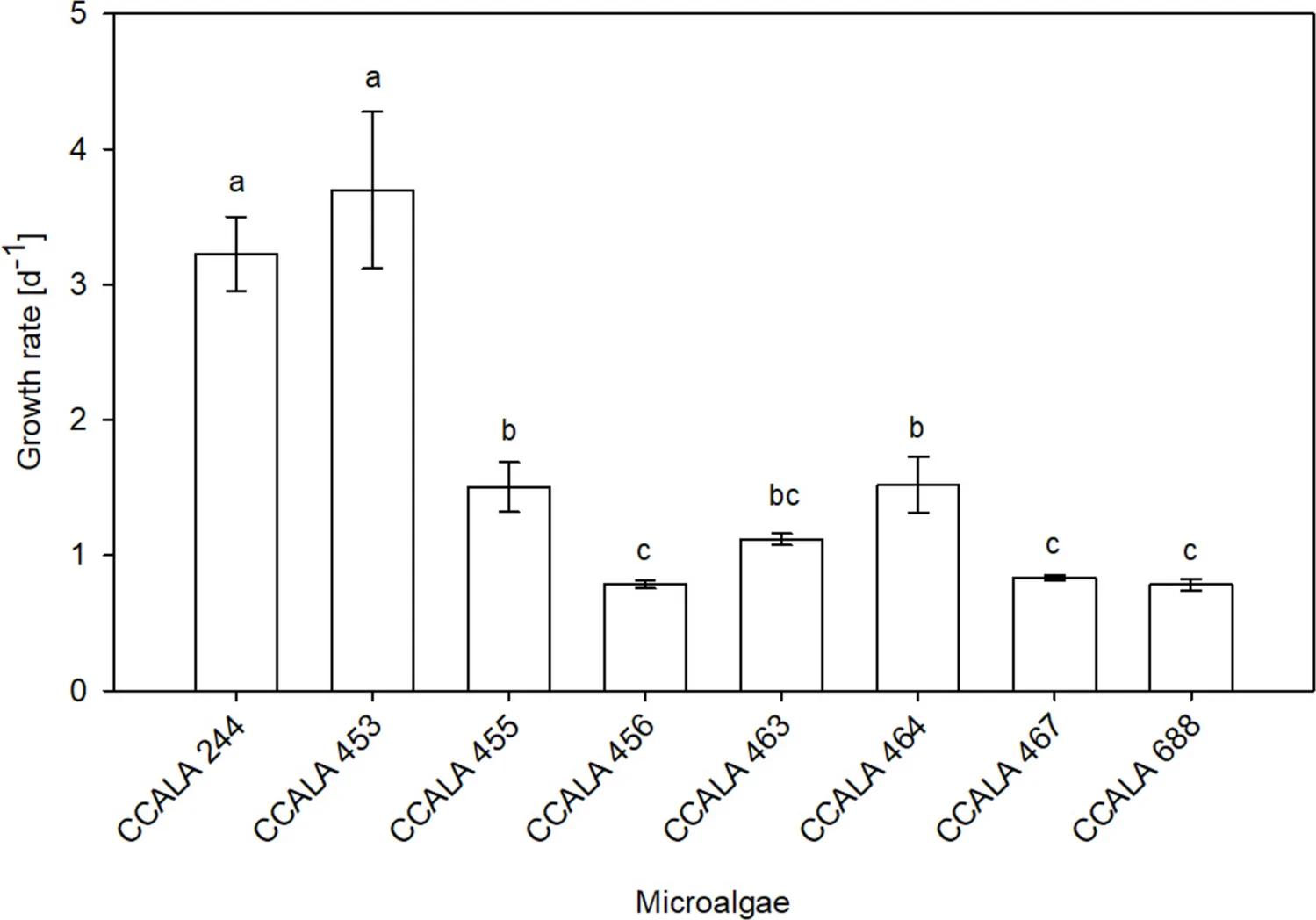
Specific growth rates [µ; day^−1^] of selected microalgae strains (see legend of Fig. 5) grown heterotrophically on 1% glucose at 30 °C for 72 h. *C. moewusii* CCALA 242 and 243 did not grow. The values are presented as a mean ± SD (*n* = 3); those columns designated by the same letter did not differ from each other

In Trial 2, the accumulation of biomass and maximum growth rate of eight heterotrophically growing microalgae were determined (Figs. 5 and 6). The strains can be divided into two groups. The first group—*C. moewusii* CCALA 244, S*. obliquus* CCALA 455, *D. communis* CCALA 463 and CCALA 464—grew faster and reached higher maximum biomass density after about 50 to 60 h of cultivation (Fig. 5a), when *C. moewusii* CCALA 244 grew the fastest. The second group of selected microalgae—*S. obliquus* CCALA 453, *S. obliquus* CCALA 456, *D. subspicatus* CCALA 688 and *D. subspicatus* CCALA 467—grew about twice as slowly and reached a lower maximum biomass density at the end of the experiment (Fig. 5b).

The specific growth rates in heterotrophically grown cultures (Fig. 6) reflected the growth patterns and were generally higher than those of photoautotrophically grown cultures (see comparison in Table 1). The highest growth rate µ = 3.70 ± 0.58 day^−1^ was determined for *S.obliquus* CCALA 453, while under the photoautotrophic regime, it was more than 10 times less. Lower growth rates of 3.23 ± 0.27 day^−1^ were found at heterotrophic conditions for *C. moewusii* strain CCALA 244. The smallest differences between growth rates under photoautotrophic and heterotrophic regimes were observed for *S. obliquus* CCALA 456 and *D. subspicatus* CCALA 467.

**Table 1:** The ratio of heterotrophic vs. photoautotrophic specific growth rates (the highest values) determined in the cultivation trials with selected microalgae strains (see legend of Fig. 1). *C. moewusii* CCALA 242 and 243 did not grow heterotrophically (nd)

Microalgae strain	Ratio of heterotrophic vs photoautotrophic growth rates
CCALA 242	nd
CCALA 243	nd
CCALA 244	7.9
CCALA 453	10.9
CCALA 455	3.1
CCALA 456	1.5
CCALA 463	3.7
CCALA 464	3.9
CCALA 467	1.7
CCALA 688	2.5

*nd* no growth detected

**Fig. 7:**
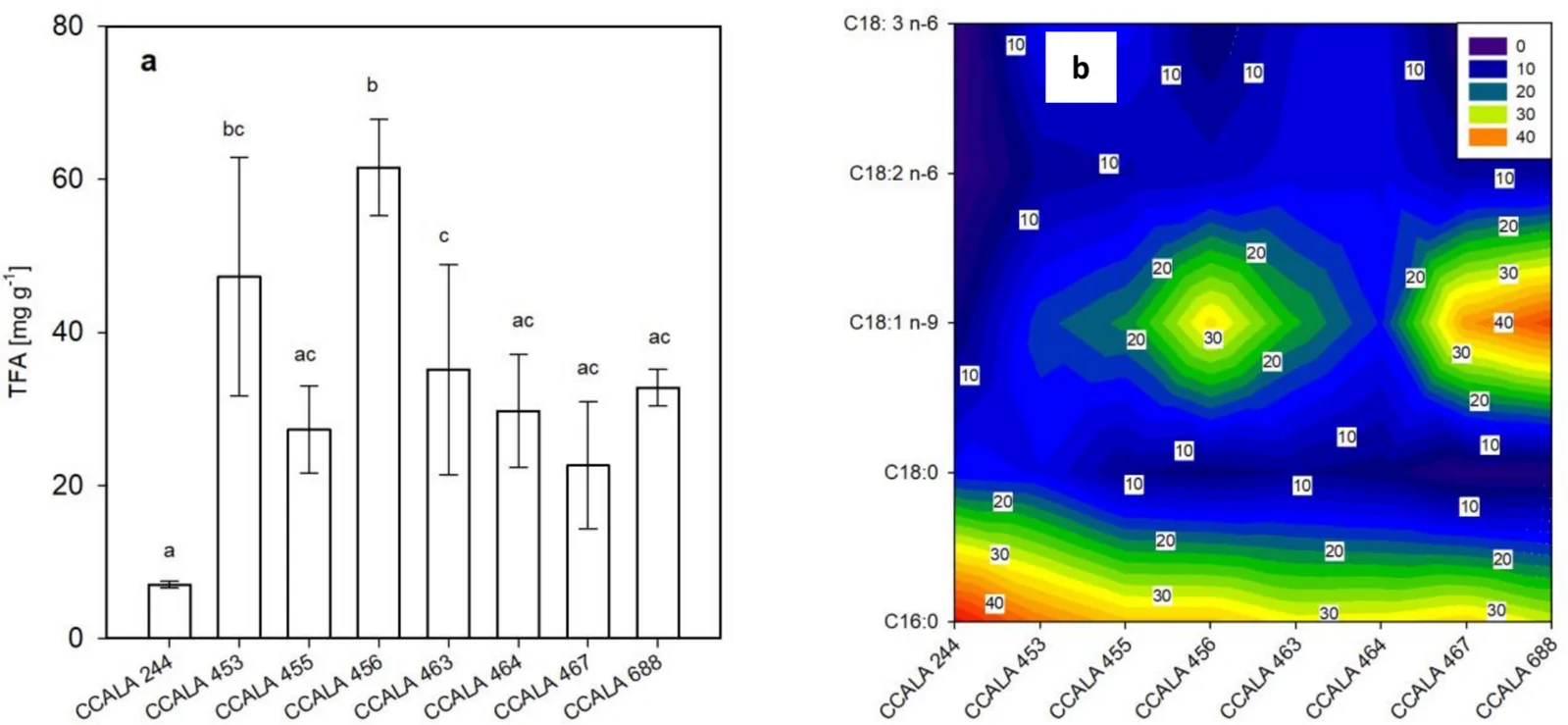
Fatty acid production of selected microalgae strains (see legend of Fig. 1) grown heterotrophically. **a** The TFA content [mg g^−1^]. **b** The concentration [% of TFA] of five selected fatty acids—palmitic PA C16:0, stearic SA C18:0, oleic OA C18:1 n-9, linoleic LA C18:2 n-6 and γ-linolenic acid GLA C18:3 n-6 were analysed in the biomass samples harvested at the end of the trial. The values are presented as a mean ± SD (*n* = 3); those designated by the same letter did not differ from each other

In the heterotrophically grown cultures, the TFA content in biomass samples was always lower as compared to photoautotrophic cultures (Fig. 7 vs. Fig. 3). The most noticeable difference in TFA content was found in *D. subspicatus* CCALA 688, as in the heterotrophically grown culture, the biomass contained 30 times less TFA compared to photoautotrophically grown biomass (see Figs. 3 and 7a). On the other hand, the quantitative representation of individual FAs relative to the TFA did not differ significantly compared to the photoautotrophic regime (see Table S1 and Table S2). The content of PA ranged from 29.6 ± 0.6 in *D. subspicatus* CCALA 688 to 47.2 ± 0.5 found in *C. moewusii* CCALA 244. SA, GLA production was even stimulated in the case of *C. moewusii* CCALA 244 and *S. obliquus* CCALA 453 in the heterotrophic regime. However, it should not be forgotten that it has been found that the TFA is significantly lower in this regime compared to photoautotrophy, as already mentioned.

## Discussion

Microalgae adapt both their growth rates and FA production to environmental conditions when temperature and light are major critical variables under non-stress conditions (Singh and Singh 2015; Zachleder et al. 2016; Maltsev et al. 2021). Here, in the presented trials, a group of microalgae naturally producing FA comparable in composition to palm oil was selected, in which the effect of the cultivation regime and varying light intensity on their growth and FA production and composition was studied while maintaining a suitable temperature. Two of the three *C. moewusii* strains (CCALA 242 and 243) favoured lower light intensities. Such low light preference was previously observed for *Chlamydomonas eugametos* (synonym to *C. moewusii*), which was routinely grown at around 100–150 µmol photons m^−2^ s^−1^(Zachleder and Van Den Ende 1992; Pröschold and Darienko 2023). On the other hand, another strain, *Chlamydomonas reinhardtii*, which is the most studied member of this genus, can grow at a wide range of light intensities from 6 to 250 µmol photons m^−2^ s^−1^ (Vítová et al. 2011) and was not light-saturated even at 500 µmol photons m^−2 s−1^ (Bialevich et al. 2022). In the present trial, the growth of *Scenedesmus* and *Desmodesmus* strains improved with increasing light intensity. The green microalgae genera, *Scenedesmus* and *Desmodesmus*, include numerous widely studied biotechnologically relevant strains. Their response to light appears to be specific based both on species/strain and growth conditions; generally, they tolerate/prefer higher growth irradiances. In several studies, the diversity of optimal light conditions of selected species of the genus *Scenedesmus* was described as dependent on specific cultivation facilities. In the literature, various *Scenedesmus* species grown in flask cultures revealed the optimal growth light intensity of 80 µmol photons m^−2^ s^−1^ (Difusa et al. 2015). Cultures of *S. obliquus* grown in flatbed reactors achieved maximum growth rates at about 150 µmol photons m^−2^ s^−1^, although they were able to tolerate or adapt to higher light (Sforza et al. 2014). Likewise, *Scenedesmus* sp. cultured in tubular photobioreactors saturated at 250 µmol photons m^−2^ s^−1^ but could maintain similar biomass yields even at 400 µmol photons m^−2^ s^−1^ (Liu et al. 2012). Also, *S. quadricauda* grown in a tubular photobioreactor favoured a higher light intensity of 500 µmol photons m^−2^ s^−1^ (Fettah et al. 2022). It can be inferred that the optimum value of irradiance intensity is determined by the set-up of the culture unit as the genus *Scenedesmus* is very adaptable.

A recent trend in microalgal research explores the potential of heterotrophic growth for less costly biomass generation and its use for FA production (Perez-Garcia et al. 2011; Bumbak et al. 2011; Sutherland and Ralph 2021). However, only a few microalgae, such as *Scenedesmus* sp., *Chlorococcum* sp., *Chlorella* sp. and *Chlamydomonas* sp., can grow heterotrophically (Chen and Johns 1996; Barros et al. 2019; Jin et al. 2020; Correia et al. 2023). This cultivation regime is usually faster, reaching high cell densities in a shorter time than in phototrophic cultivation, as the growth density is not limited by light (Carone et al. 2019). This point is advantageous for further downstream processing of the microalgae biomass and can reduce production costs despite the addition of an organic carbon source. Of the ten pre-selected microalgae in this study, eight strains were able to grow heterotrophically (and even faster) than those in photoautotrophic cultures. The highest growth rate was determined under the heterotrophic regime for *S.obliquus* CCALA 453 while in the photoautotrophic regime, it was more than one order of magnitude lower. As a matter of fact, a much lower growth rate of *S. obliquus* of 0.7 day^−1^ was determined by Silkina et al. (2025). The specific growth rate of 1.03 day^−1^ determined by Jin et al. (2020) for *S. acuminatus* was slightly close to the value we found for *D. communis* CCALA 463.

In general, in photoautotrophic cultures at a high light intensity, SFA and MUFA accumulate while the amounts of PUFA decrease (Maltsev et al. 2021). Microalgae downregulate PUFA, as a protective mechanism, to reduce membrane fluidity and protect cell integrity from photooxidative stress (Van Wagenen et al. 2012; Morales-Sánchez et al. 2020). In this study, the largest difference in SFA production at the lowest and highest light intensity was observed for *D. subspicatus* 467 and *D. subspicatus* 688. At the same time, at the highest light intensity, the largest increase in MUFA was observed for *S.obliquus* strains CCALA 453 and CCALA 455, and *D. subspicatus* CCALA 467. In *C. moewusii* CCALA 244, the content of PA, SA, OA and LA detected was comparable regardless of the cultivation regime. The same result was found by El-Sheekh (1993) for *C. reindhardtii*. However, all *Chlamydomonas* strains grown in the photoautotrophic regime and studied in this work produced up to a threefold higher amount of PA and about 1.5-fold more SA, respectively. The content of individual FAs in the *C. moewusii* strains CCALA 242 and CCALA 243 was comparable to the amount reported by Zheng et al. (2022) for *Chlamydomonas reinhardtii*. Moreover, in the selected *Scenedesmus* and *Desmodesmus* species, the amount of PA was comparable to data obtained for *Scenedesmus bijugus* (Salama et al. 2013; Minhas et al. 2020). On the other hand, the difference is evident in the SA content, as *S.bijugus* contains almost none (Salama et al. 2013; Minhas et al. 2020). While palm oil contains negligible amounts of GLA (Mancini et al. 2015), certain microalgae from the selected group in this study are capable of producing remarkably high levels of GLA, reaching up to 33% of TFA. This exceptional capacity determines microalgae as a superior and sustainable source of essential FAs, making them particularly valuable for biotechnological applications. Continuous illumination used in this study stimulated the production of OA and LA in selected *Scenedesmus* and *Desmodesmus* strains, as the measured values of these two FAs were 2.0–3.5-fold higher (depending on the light intensity) than those found by Minhas et al. (2020), who alternated light and dark regimes. Although the TFA content was different in the biomass of the selected set of microalgae regardless of the cultivation regime, the content of the total amount of individual FAs was identical. The low content of TFA in heterotrophically grown microalgae can be attributed to the rapid growth during a short cultivation period (72 h) when microalgae produce high biomass but do not accumulate high-value products such as lipids (Udayan et al. 2022).

In general, the highest TFA content was found in the biomass of *S. obliquus* CCALA 455 and *D. subspicatus* CCALA 467 grown photoautotrophically, making them interesting for potential commercial interest, as these numbers are about 1.6–1.8-fold higher compared to palm oil (36%) (Mata et al. 2010; Pugliese et al. 2020). For comparison, the amounts of selected FAs (PA, OA and LA) determined in *D. subspicatus* CCALA 467 were closest to those in palm oil (Mancini et al. 2015), with the added benefit of significant GLA production. However, the production cost of 1 L of microalgae oil is still significantly higher (up to 3.5 USD depending on the cultivation unit used) (Valdovinos-García et al. 2022) than that of palm oil (app. 0.77 USD L^−1^) (O’Neill 2025). On the other hand, microalgae represent an advantageous alternative not only in terms of the production of FAs contained in palm oil, but also thanks to their significantly lower land requirements, namely 2.79–0.33 ha, depending on productivity compared to the requirements for palm plantations (5.20 ha). Consequently, microalgae oil production reaches 11.00–93.50 L/ha/year compared to 5.90 L of palm oil/ha/year in the case of palm trees (Adi Sasongko and Noguchi 2015).

Despite being inexpensive, the production of palm oil is burdened by its significant environmental impact. Deforestation before the planting of palm trees has been a serious global issue (Waghmare et al. 2018). The advantage of using selected microalgae species/strains that can produce a very similar FA composition to palm oil has to be further considered and investigated, as optimisation of culture conditions may result in the overproduction of selected FAs compared to standard culture conditions. Moreover, the production costs can be further decreased by using wastewater or seawater (Raheem et al. 2018). In general, technological improvements in microalgae culturing and processing are steadily decreasing costs, reinforcing its role as a promising alternative to replace palm oil (Rafa et al. 2021; Valdovinos-García et al. 2022; Stănescu et al. 2024; Tripathi et al. 2024).

## Supplementary Information

Below is the link to the electronic supplementary material.

ESM 1 (DOCX 24.2 KB)

ESM 2 (DOCX 16.7 KB)

ESM 3 (DOCX 15.5 KB)

## Data Availability

The datasets presented in this study are available from the corresponding authors upon reasonable request. The data are not publicly available without the permission of all co-authors.
