# Supplementary material for: Microalgae as a sustainable alternative to palm oil: fatty acid profiles under photoautotrophic and heterotrophic growth
Source: Appl Microbiol Biotechnol. 2026 Jan 12;110:17. doi: 10.1007/s00253-025-13682-0 (PMC12799715; doi:10.1007/s00253-025-13682-0)
Supplement: Supplementary file 2 [file 253_2025_13682_MOESM2_ESM.docx]

|  | **CCALA 244** | **CCALA 453** | **CCALA 455** | **CCALA 456** | **CCALA 463** | **CCALA 464** | **CCALA 467** | **CCALA 688** |
| --- | --- | --- | --- | --- | --- | --- | --- | --- |
| **C12:0** | 0.3 ± 0.0 ^a^ | 0.3 ± 0.2 ^a^ | 0.1 ± 0.0 ^a^ | 0 ^a^ | 0.2 ± 0.2 ^a^ | 0.2 ± 0.1 ^a^ | 0 ^a^ | 0 ^a^ |
| **C14:0** | 5.0 ± 0.8 ^a^ | 5.5 ± 0.0 ^a^ | 1.4 ± 0.0 ^b^ | 0 ^c^ | 0.8 ± 0.6 ^b^ | 1.4 ± 0.0 ^b^ | 3.9 ± 0.0 | 0 ^c^ |
| **C16:0** | 47.2 ± 0.5 ^a^ | 39.4 ± 5.5 ^a^ | 34.7 ± 4.9 ^a^ | 34.7 ± 9.6 ^a^ | 31.1 ± 9.9 ^a^ | 31.5 ± 6.3 ^a^ | 33.4 ± 0.3 ^a^ | 29.6 ± 0.6 ^a^ |
| **C16:1 n-7** | 15.7 ± 1.8 ^a^ | 18.3 ± 7.0 ^a^ | 5.2 ± 0.6 ^b^ | 6.6 ± 1.5 | 6.6 ± 1.4 ^b^ | 5.9 ± 0.9 ^b^ | 8.3 ± 0.4 ^b^ | 7.8 ± 1.0 ^b^ |
| **C16:2** | 1.2 ± 0.3 ^a^ | 2.5 ± 1.7 ^a^ | 1.6 ± 0.1 ^a^ | 3.2 ± 0.8 ^a^ | 3.7 ± 0.6 ^b^ | 4.4 ± 0.6 ^b^ | 1.5 ± 0.3 ^a^ | 2.3 ± 0.6 ^a^ |
| **C16:3** | 0.8 ± 0.5 ^a^ | 1.3 ± 0.3 ^b^ | 7.0 ± 0.9 ^c^ | 2.8 ± 0.2 ^b^ | 4.6 ± 0.8 ^d^ | 5.3 ± 0.7 ^d^ | 1.5 ± 0.2 ^b^ | 1.5 ± 0.8 ^b^ |
| **C18:0** | 14.8 ± 1.5 ^a^ | 13.8 ± 5.0 ^a^ | 7.9 ± 1.6 ^b^ | 6.9 ± 0.0 ^b^ | 8.0 ± 3.0 ^b^ | 6.7 ± 1.2 ^b^ | 3.1 ± 0.3 ^c^ | 3.5 ± 0.2 ^c^ |
| **C18:1 n-9** | 6.0 ± 1.7 ^a^ | 17.3 ± 3.7 ^b^ | 20.6 ± 2.4 ^b^ | 32.9 ± 0.2 ^c^ | 22.4 ± 1.0 ^b^ | 15.8 ± 0.2 ^b^ | 37.5 ± 1.0 ^c^ | 43.2 ± 0.1 ^d^ |
| **C18:1 n-7** | 3.6 ± 0.2 ^a^ | 0 ^b^ | 0.1 ± 0.1 ^b^ | 0.1 ± 0.1 ^b^ | 0.3 ± 0.5 ^b^ | 5.3 ± 1.1 ^a^ | 0.1 ± 0.1 ^b^ | 0.6 ± 0.3 ^b^ |
| **C18:2 n-6** | 0.9 ± 1.3 ^a^ | 8.5 ± 0.6 ^b^ | 9.9 ± 0.1 ^b^ | 10.9 ± 0.4 ^b^ | 13.2 ± 4.5 ^b^ | 13.4 ± 1.5 ^b^ | 9.0 ± 1.3 ^b^ | 8.9 ± 1.6 ^b^ |
| **C18:3 n-6** | 0 ^a^ | 14.5 ± 2.0 ^b^ | 14.7 ± 1.2 ^b^ | 6.0 ± 1.9 ^c^ | 11.9 ± 0.6 ^b^ | 12.7 ± 0.3 ^b^ | 3.4 ± 0.4 ^c^ | 3.7 ± 1.5 ^c^ |
| **C18:3 n-3** | 0.4 ± 0.4 ^a^ | 1.7 ± 0.4 ^b^ | 2.6 ± 0.1 ^b^ | 1.7 ± 0.8 ^b^ | 1.6 ± 0.1 ^b^ | 1.9 ± 0.2 ^b^ | 1.3 ± 0.3 ^b^ | 1.7 ± 0.5 ^b^ |
| **C20:0** | 0.1 ± 0.1 ^a^ | 0.7 ± 0.2 ^ab^ | 0.3 ± 0.1 ^ab^ | 0.6 ± 0.1 ^ab^ | 0.9 ± 0.8 ^ab^ | 1.2 ± 0.3 ^b^ | 1.0 ± 0.2 ^ab^ | 1.2 ± 0.2 ^b^ |

**Table S2** The content of individual fatty acids in selected microalgae strains (see the list in the legend of Fig. 1) during heterotrophic cultivation in the presence of 1 % of glucose in the cultivation medium. *C. moewusii* CCALA 242 and 243 did not grow. Statistical analysis was performed between individual microalgae for given fatty acid. The values correspond to the content measured at the end of the experiment. The values are presented as a mean (n = 3) ± SD.
