# Supplementary material for: Microalgae as a sustainable alternative to palm oil: fatty acid profiles under photoautotrophic and heterotrophic growth
Source: Appl Microbiol Biotechnol. 2026 Jan 12;110:17. doi: 10.1007/s00253-025-13682-0 (PMC12799715; doi:10.1007/s00253-025-13682-0)
Supplement: Supplementary file 3 [file 253_2025_13682_MOESM3_ESM.docx]

| **Productivity/Microalgae** | **LI = 50 µmol m^-2^ s^-1^** | | | | | | | | | |
| --- | --- | --- | --- | --- | --- | --- | --- | --- | --- | --- |
|  | **CCALA 242** | **CCALA 243** | **CCALA 244** | **CCALA 453** | **CCALA 455** | **CCALA 456** | **CCALA 463** | **CCALA 464** | **CCALA 467** | **CCALA 688** |
| **P_V_** | 0.13 | 0.14 | 0.06 | 0.31 | 0.28 | 0.25 | 0.28 | 0.22 | 0.29 | 0.21 |
| **P_A_** | 0.76 | 0.85 | 0.34 | 1.81 | 1.62 | 1.49 | 1.64 | 1.31 | 1.71 | 1.21 |
|  | **LI = 100 µmol m^-2^ s^-1^** | | | | | | | | | |
| **P_V_** | 0.17 | 0.07 | 0.08 | 0.56 | 0.50 | 0.45 | 0.38 | 0.44 | 0.44 | 0.35 |
| **P_A_** | 0.99 | 0.42 | 0.47 | 3.32 | 2.96 | 2.67 | 2.23 | 2.59 | 2.56 | 2.06 |
|  | **LI = 200 µmol m^-2^ s^-1^** | | | | | | | | | |
| **P_V_** | 0.11 | nd | 0.08 | 0.57 | 0.76 | 0.64 | 0.57 | 0.56 | 0.50 | 0.41 |
| **P_A_** | 0.67 | nd | 0.47 | 3.38 | 4.46 | 3.77 | 3.37 | 3.31 | 2.94 | 2.40 |
|  | **LI = 400 µmol m^-2^ s^-1^** | | | | | | | | | |
| **P_V_** | 0.05 | nd | 0.11 | 0.69 | 0.90 | 0.57 | 0.58 | 0.58 | 0.69 | 0.59 |
| **P_A_** | 0.31 | nd | 0.64 | 4.10 | 5.31 | 3.38 | 3.44 | 3.44 | 4.06 | 3.45 |

**Table S3** Volumetric (P_V_) [g L^-1^ d^-1^] and areal (P_A_) [g m^-2^ d^-1^] productivity of selected microalgae (see the list in the legend of Fig. 1) at four different light intensities during light optimization in photoautotrophic mode. *C. moewusii* CCALA 243 did not grow at LI = 200 and 400 µmol m^-2^ s^-1^.

nd = not determined
