# Supplementary material for: Microalgae as a sustainable alternative to palm oil: fatty acid profiles under photoautotrophic and heterotrophic growth
Source: Appl Microbiol Biotechnol. 2026 Jan 12;110(1):17. doi: 10.1007/s00253-025-13682-0 (PMC12799715; doi:10.1007/s00253-025-13682-0)
Supplement: Supplementary file 1 — (DOCX 24.2 KB) [file 253_2025_13682_MOESM1_ESM.docx]

**Table S1** The content of individual fatty acids in selected microalgae strains (see the list in the legend of Fig. 1) at four different light intensities during light optimization in photoautotrophic mode. Statistical analysis was performed between the rows. The values are presented as a mean (n = 3) ± SD.

| **Fatty acid/Microalgae** | **LI = 50 µmol m^-2^ s^-1^** | | | | | | | | | |
| --- | --- | --- | --- | --- | --- | --- | --- | --- | --- | --- |
|  | **CCALA 242** | **CCALA 243** | **CCALA 244** | **CCALA 453** | **CCALA 455** | **CCALA 456** | **CCALA 463** | **CCALA 464** | **CCALA 467** | **CCALA 688** |
| **C12:0** | 0^a^ | 0^a^ | 0.3 ± 0.1^a^ | 0.1 ± 0.0^a^ | 0.1 ± 0.0^a^ | 0.2 ± 0.2^a^ | 0.1 ± 0.0^a^ | 0.3 ± 0.1^a^ | 0.1 ± 0.0^a^ | 0^a^ |
| **C14:0** | 0.7 ± 0.1^a^ | 0.4 ± 0.0^a^ | 2.1 ± 0.1^b^ | 0.8 ± 0.5^a^ | 1.1 ± 1.1^a^ | 0.6 ± 0.2^a^ | 0.1 ± 0.0^a^ | 1.2 ± 0.1^b^ | 0.7 ± 0.4^a^ | 0.5 ± 0.2^a^ |
| **C16:0** | 22.9 ± 0.7^a^ | 25.8 ± 0.7^a^ | 31.9 ± 1.9^b^ | 24.6 ± 1.9^a^ | 35.3 ± 1.4^b^ | 27.1 ± 2.9^a^ | 38.4 ± 0.6^b^ | 32.7 ± 1.4^b^ | 20.5 ± 1.6^a^ | 24.9 ± 0.5^a^ |
| **C16:1 n-7** | 0.7 ± 0.1^a^ | 0.6 ± 0.0^a^ | 1.5 ± 0.4^b^ | 3.6 ± 0.5^c^ | 1.9 ± 0.1^b^ | 6.6 ± 0.8^d^ | 1.6 ± 0.2^b^ | 3.3 ± 0.4^c^ | 6.3 ± 0.2^d^ | 3.2 ± 0.2^c^ |
| **C16:2** | 2.8 ± 0.4^a^ | 1.4 ± 0.3^b^ | 2.3 ± 0.2^a^ | 2.7 ± 0.1^a^ | 0.8 ± 0.^b^1 | 2.6 ± 0.6^a^ | 2.3 ± 0.2^a^ | 3.5 ± 0.5^a^ | 6.6 ± 0.6^c^ | 0^d^ |
| **C16:3** | 3.5 ± 0.3 | 2.9 ± 0.1 | 2.9 ± 0.1 | 8.3 ± 0.5 | 4.4 ± 0.6 | 6.0 ± 2.4 | 2.6 ± 0.9 | 8.8 ± 0.4 | 4.8 ± 0.8 | 0 |
| **C18:0** | 11.5 ± 1.3^a^ | 10.2 ± 0.6^a^ | 12.9 ± 3.4^a^ | 6.7 ± 0.1^b^ | 7.5 ± 0.6^b^ | 2.7 ± 0.3^c^ | 3.8 ± 0.3^c^ | 3.6 ± 0.^c^3 | 6.4 ± 0.1^b^ | 12.3 ± 0.8^a^ |
| **C18:1 n-9** | 11.4 ± 3.5^a^ | 13.9 ± 1.6^a^ | 9.6 ± 1.2^a^ | 18.1 ± 1.4^b^ | 20.9 ± 1.4^b^ | 29.4 ± 4.6^c^ | 18.8 ± 4.7^b^ | 16.3 ± 1.1^b^ | 21.0 ± 0.8^b^ | 16.5 ± 1.9^b^ |
| **C18:1 n-7** | 9.0 ± 0.1^a^ | 8.5 ± 0.2^a^ | 0^b^ | 0^b^ | 1.0 ± 0.2^b^ | 0.2 ± 0.1^b^ | 18.6 ± 4.4^c^ | 0^b^ | 0.6 ± 0.1^b^ | 1.1 ± 0.1^b^ |
| **C18:2 n-6** | 6.4 ± 1.2^a^ | 4.5 ± 0.9^a^ | 6.3 ± 0.^a^3 | 17.0 ± 0.9^b^ | 12.0 ± 1.2^b^ | 15.7 ± 2.4^b^ | 14.6 ± 2.5^b^ | 14.3 ± 1.1^b^ | 20.0 ± 0.3^b^ | 15.0 ± 0.8^b^ |
| **C18:3 n-6** | 30.8 ± 3.6^a^ | 31.3 ± 2.4^a^ | 26.9 ± 1.4^a^ | 16.4 ± 0.1^b^ | 13.9 ± 0.7^b^ | 6.7 ± 2.3^c^ | 8.2 ± 2.4^c^ | 13.2 ± 1.7^b^ | 11.2 ± 1.1^b^ | 3.1 ± 0.2^c^ |
| **C18:3 n-3** | 0.1 ± 0.0^a^ | 0.2 ± 0.0^a^ | 0.1 ± 0.1^a^ | 0.2 ± 0.0^a^ | 0.5 ± 0.1^a^ | 0.3 ± 0.0^a^ | 0.7 ± 0.1^a^ | 0.9 ± 0.0^a^ | 0.1 ± 0.1^a^ | 20.7 ± 1.1^b^ |
| **C20:0** | 0.2 ± 0.1^a^ | 0.2 ± 0.0^a^ | 1.0 ± 0.1^b^ | 1.5 ± 0.1^b^ | 0.7 ± 0.3^a^ | 2.0 ± 0.3^b^ | 1.1 ± 0.3^b^ | 1.9 ± 0.0^b^ | 1.7 ± 0.0^b^ | 0^a^ |
|  | **LI = 100 µmol m^-2^ s^-1^** | | | | | | | | | |
| **C12:0** | 0^a^ | 0^a^ | 0.1 ± 0.1^a^ | 0.1 ± 0.0^a^ | 0^a^ | 0.1 ± 0.0^a^ | 0.1 ± 0.0^a^ | 0.2 ± 0.1^a^ | 0^a^ | 0^a^ |
| **C14:0** | 0.4 ± 0.1^a^ | 0.8 ± 0.1^a^ | 1.6 ± 0.2^b^ | 0.8 ± 0.2^a^ | 0.4 ± 0.2^a^ | 0.4 ± 0.2^a^ | 0.1 ± 0.0^a^ | 0.5 ± 0.1^a^ | 0.3 ± 0.1^a^ | 3.1 ± 0.2^c^ |
| **C16:0** | 22.6 ± 0.9^a^ | 27.3 ± 1.0^a^ | 28.6 ± 1.3^a^ | 28.9 ± 1.8^a^ | 33.8 ± 0.7^b^ | 28.9 ± 1.6^a^ | 40.2 ± 2.4^c^ | 34.6 ± 1.3^b^ | 30.5 ± 1.5^a^ | 27.7 ± 0.6^a^ |
| **C16:1 n-7** | 0.6 ± 0.0^a^ | 1.2 ± 0.1^a^ | 1.3 ± 0.2^a^ | 2.1 ± 0.4^b^ | 1.9 ± 0.2^b^ | 4.0 ± 0.4^c^ | 1.6 ± 0.2^a^ | 2.0 ± 0.1^b^ | 4.8 ± 1.1^c^ | 2.4 ± 0.0^b^ |
| **C16:2** | 1.9 ± 0.1^a^ | 1.4 ± 0.0^a^ | 2.6 ± 0.4^a^ | 3.1 ± 0.2^a^ | 0.9 ± 0.1^a^ | 2.7 ± 0.4^a^ | 2.0 ± 0.2^a^ | 1.9 ± 0.1^a^ | 3.6 ± 0.5^a^ | 0^b^ |
| **C16:3** | 2.9 ± 0.2^a^ | 1.9 ± 0.2^a^ | 3.6 ± 0.^a^4 | 1.9 ± 0.5^a^ | 3.7 ± 0.4^a^ | 3.8 ± 0.9^a^ | 2.7 ± 0.2^a^ | 3.8 ± 0.6^a^ | 1.7 ± 0.1^a^ | 0^b^ |
| **C18:0** | 9.1 ± 0.5^a^ | 9.1 ± 0.2^a^ | 14.8 ± 1.4^b^ | 6.7 ± 1.3^a^ | 7.6 ± 0.1^a^ | 4.0 ± 0.4^c^ | 4.4 ± 0.6^c^ | 7.5 ± 0.9^d^ | 6.5 ± 0.5^d^ | 23.5 ± 0.7^e^ |
| **C18:1 n-9** | 19.0 ± 1.3^a^ | 17.2 ± 0.9^a^ | 9.2 ± 0.3^b^ | 27.2 ± 3.5^c^ | 30.9 ± 2.1^c^ | 32.1 ± 0.6^c^ | 21.6 ± 1.5^a^ | 20.0 ± 0.7^a^ | 28.3 ± 2.5^c^ | 0.8 ± 0.0^d^ |
| **C18:1 n-7** | 10.6 ± 0.1^a^ | 9.6 ± 0.6^a^ | 0^b^ | 0^b^ | 0.2 ± 0.1^b^ | 0.2 ± 0.0^b^ | 0^b^ | 0^b^ | 0.1 ± 0.1^b^ | 13.9 ± 0.2^a^ |
| **C18:2 n-6** | 6.0 ± 0.3^a^ | 5.2 ± 0.0^a^ | 7.1 ± 1.3^a^ | 16.9 ± 0.6^b^ | 11.3 ± 0.4^c^ | 15.3 ± 0.3^b^ | 15.6 ± 0.2^b^ | 12.5 ± 0.4^c^ | 15.6 ± 1.^b^9 | 2.4 ± 0.0^d^ |
| **C18:3 n-6** | 26.6 ± 1.4^a^ | 26.0 ± 1.0^a^ | 29.9 ± 1.5^a^ | 11.0 ± 1.7^b^ | 8.8 ± 1.5^b^ | 6.9 ± 1.2^b^ | 10.2 ± 1.1^b^ | 15.2 ± 1.1^c^ | 7.1 ± 1.9^b^ | 16.3 ± 0.4^c^ |
| **C18:3 n-3** | 0.1 ± 0.0^a^ | 0.3 ± 0.0^a^ | 0^a^ | 0.2 ± 0.0^a^ | 0.2 ± 0.0^a^ | 0.2 ± 0.0^a^ | 0.6 ± 0.1^a^ | 0.4 ± 0.0^a^ | 0.1 ± 0.0 | 0.1 ± 0.0 |
| **C20:0** | 0.1 ± 0.0 ^a^ | 0.1 ± 0.1 ^a^ | 1.0 ± 0.1 ^b^ | 1.0 ± 0.1 ^b^ | 0.3 ± 0.2 ^a^ | 1.5 ± 0.1 ^b^ | 0.9 ± 0.1 ^b^ | 1.2 ± 0.1 ^b^ | 1.5 ± 0.4 ^b^ | 0.1 ± 0.1 ^a^ |

|  | **LI = 200 µmol m^-2^ s^-1^** | | | | | | | | | |
| --- | --- | --- | --- | --- | --- | --- | --- | --- | --- | --- |
| **C12:0** | 0 ^a^ | nd | 0.1 ± 0.0 ^a^ | 0 ^a^ | 0 ^a^ | 0 ^a^ | 0.1 ± 0.1 ^a^ | 0.1 ± 0.0 ^a^ | 0 ^a^ | 0 ^a^ |
| **C14:0** | 0.4 ± 0.2 ^a^ | nd | 2.5 ± 0.8 ^b^ | 0.6 ± 0.1 ^a^ | 0.3 ± 0.0 ^a^ | 0.4 ± 0.2 ^a^ | 0.4 ± 0.3 ^a^ | 0.5 ± 0. ^a^ 1 | 0.3 ± 0.1 ^a^ | 0.4 ± 0.1 ^a^ |
| **C16:0** | 23.9 ± 0.1^a^ | nd | 43.2 ± 4.4^b^ | 28.8 ± 0.8^a^ | 35.1 ± 0.6^c^ | 29.2 ± 0.2^a^ | 37.7 ± 1.5^c^ | 33.3 ± 1.1^c^ | 36.1 ± 1.2^c^ | 36.5 ± 2.4^c^ |
| **C16:1 n-7** | 0.7 ± 0.2 ^a^ | nd | 1.2 ± 1.1 ^a^ | 1.7 ± 0.3 ^b^ | 1.5 ± 0.1 ^b^ | 3.2 ± 1.1 ^c^ | 1.8 ± 0.2 ^b^ | 2.0 ± 0.1 ^b^ | 3.3 ± 1.1 ^c^ | 2.0 ± 0.5 ^b^ |
| **C16:2** | 1.7 ± 0.1 ^a^ | nd | 1.3 ± 0. ^a^ 5 | 3.4 ± 0.2 ^b^ | 1.0 ± 0.1 ^a^ | 2.7 ± 0.1 ^c^ | 1.5 ± 0.2 ^a^ | 1.7 ± 0.2 ^a^ | 2.1 ± 0.2 ^c^ | 0 ^d^ |
| **C16:3** | 2.5 ± 0.3 ^a^ | nd | 2.3 ± 0.5 ^a^ | 1.5 ± 0.3 ^b^ | 2.8 ± 0.2 ^a^ | 2.4 ± 0.7 ^a^ | 1.8 ± 0.1 ^b^ | 3.3 ± 0.3 ^a^ | 1.5 ± 0.0 ^b^ | 0 ^c^ |
| **C18:0** | 8.0 ± 0.4 ^a^ | nd | 9.5 ± 2.2 ^a^ | 5.6 ± 0.3 ^b^ | 7.5 ± 0.4 ^b^ | 3.9 ± 0.5 ^c^ | 7.0 ± 0.5 ^b^ | 8.4 ± 0.7 ^a^ | 5.6 ± 1.0 ^b^ | 7.9 ± 0.4 ^ab^ |
| **C18:1 n-9** | 20.6 ± 1.0^a^ | nd | 10.7 ± 1.3^b^ | 30.9 ± 1.5^c^ | 35.7 ± 1.4^c^ | 34.8 ± 2.4^c^ | 27.2 ± 0.1^c^ | 22.3 ± 1.4^a^ | 30.1 ± 2.9^c^ | 25.3 ± 1.0^a^ |
| **C18:1 n-7** | 12.1 ± 0.6^a^ | nd | 0 ^b^ | 0 ^b^ | 0 ^b^ | 0.2 ± 0.1 ^b^ | 0 ^b^ | 0 ^b^ | 0.1 ± 0.2 ^b^ | 0.2 ± 0.1 ^b^ |
| **C18:2 n-6** | 5.7 ± 0.6 ^a^ | nd | 3.9 ± 0.6 ^a^ | 16.9 ± 0.7^b^ | 9.9 ± 0.3 ^c^ | 15.6 ± 1.1^b^ | 11.9 ± 0.8^b^ | 12.5 ± 0.7^b^ | 13.7 ± 0.6^b^ | 14.6 ± 0.2^b^ |
| **C18:3 n-6** | 24.3 ± 0.7^a^ | nd | 24.2 ± 4.3^a^ | 9.4 ± 0.6 ^b^ | 6.0 ± 0.5 ^c^ | 5.6 ± 0.6 ^c^ | 9.9 ± 0.6 ^b^ | 14.5 ± 0.9^d^ | 5.8 ± 2.0 ^c^ | 1.6 ± 0.1 ^e^ |
| **C18:3 n-3** | 0.1 ± 0.0 ^a^ | nd | 0.2 ± 0.1 ^a^ | 0.2 ± 0.0 ^a^ | 0.1 ± 0.0 ^a^ | 0.2 ± 0.0 ^a^ | 0.3 ± 0.1 ^a^ | 0.3 ± 0.0 ^a^ | 0.1 ± 0.0 ^a^ | 9.9 ± 1.0 ^a^ |
| **C20:0** | 0 ^a^ | nd | 0.9 ± 0.3 ^a^ | 0.9 ± 0.1 ^a^ | 0.1 ± 0.0 ^a^ | 1.8 ± 0.3 ^b^ | 0.6 ± 0.3 ^a^ | 1.0 ± 0.1 ^b^ | 1.2 ± 0.1 ^b^ | 0.2 ± 0.0 ^a^ |
|  | **LI = 400 µmol m^-2^ s^-1^** | | | | | | | | | |
| **C12:0** | 0 ^a^ | nd | 0.1 ± 0.0 ^a^ | 0 ^a^ | 0 ^a^ | 0.1 ± 0.0 ^a^ | 0 ^a^ | 0.1 ± 0.0 ^a^ | 0 ^a^ | 0 ^a^ |
| **C14:0** | 0.6 ± 0.5 ^a^ | nd | 3.5 ± 0.4 ^b^ | 0.5 ± 0.2 ^a^ | 0.3 ± 0.1 ^a^ | 0.4 ± 0.2 ^a^ | 0.3 ± 0.1 ^a^ | 0.7 ± 0.3 ^a^ | 0.3 ± 0.0 ^a^ | 0.4 ± 0.0 ^a^ |
| **C16:0** | 23.6 ± 0.7^a^ | nd | 42.3 ± 1.5^b^ | 30.3 ± 0.1^c^ | 33.7 ± 1.8^c^ | 29.1 ± 1.2^c^ | 34.1 ± 0.3^c^ | 35.4 ± 0.4^c^ | 37.6 ± 1.0^c^ | 37.7 ± 0.9^c^ |
| **C16:1 n-7** | 0.4 ± 0.6 ^a^ | nd | 2.1 ± 0.2 ^b^ | 1.0 ± 0.1 ^c^ | 1.3 ± 0.0 ^c^ | 2.6 ± 0.6 ^b^ | 1.6 ± 0.1 ^c^ | 2.0 ± 0.0 ^b^ | 3.2 ± 0.4 ^b^ | 2.1 ± 0.2 ^b^ |
| **C16:2** | 1.9 ± 0.1 ^a^ | nd | 1.8 ± 0.2 ^a^ | 3.2 ± 0.1 ^b^ | 1.0 ± 0.1 ^a^ | 2.2 ± 0.3 ^a^ | 1.3 ± 0.0 ^a^ | 1.2 ± 0.1 ^a^ | 2.1 ± 0.1 ^a^ | 0 ^c^ |
| **C16:3** | 2.1 ± 0.1 ^a^ | nd | 2.6 ± 0.0 ^a^ | 0.7 ± 0.0 ^b^ | 2.6 ± 0.1 ^a^ | 1.5 ± 0.2 ^c^ | 2.2 ± 0.1 ^a^ | 2.8 ± 0.2 ^a^ | 1.4 ± 0.0 ^c^ | 0 ^b^ |
| **C18:0** | 8.5 ± 0.8 ^a^ | nd | 9.0 ± 1.7 ^a^ | 4.8 ± 0.4 ^b^ | 7.6 ± 0.1 ^c^ | 4.1 ± 0.1 ^b^ | 8.0 ± 0.0 ^a^ | 9.0 ± 0.3 ^a^ | 5.2 ± 0.5 ^b^ | 8.0 ± 0.3 ^a^ |
| **C18:1 n-9** | 16.8 ± 0.5^a^ | nd | 11.6 ± 1.7^b^ | 34.4 ± 1.8^c^ | 37.4 ± 0.7^c^ | 37.7 ± 1.1^c^ | 28.0 ± 0.4^d^ | 24.2 ± 1.5^e^ | 30.1 ± 0.8^d^ | 22.3 ± 0.7^e^ |
| **C18:1 n-7** | 11.9 ± 1.0^a^ | nd | 0 ^b^ | 0 ^b^ | 0 ^b^ | 0.3 ± 0.0 ^b^ | 0 ^b^ | 0 ^b^ | 0 ^b^ | 0.2 ± 0.0 ^b^ |
| **C18:2 n-6** | 6.5 ± 0.2 ^a^ | nd | 4.9 ± 0.9 ^a^ | 15.6 ± 0.5^b^ | 10.5 ± 0.9^b^ | 14.7 ± 0.6^b^ | 13.1 ± 0.4^b^ | 10.4 ± 0.4^b^ | 14.3 ± 0.2^b^ | 16.0 ± 0.5^b^ |
| **C18:3 n-6** | 27.1 ± 0.0^a^ | nd | 21.5 ± 1.1^a^ | 8.4 ± 0.9 ^b^ | 5.2 ± 0.1 ^c^ | 5.3 ± 0.9 ^c^ | 10.7 ± 0.1^b^ | 13.4 ± 0.5^d^ | 4.5 ± 0.2 ^c^ | 1.8 ± 0.1 ^e^ |
| **C18:3 n-3** | 0.3 ± 0.1 ^a^ | nd | 0.1 ± 0.1 ^a^ | 0.1 ± 0.0 ^a^ | 0.1 ± 0.0 ^a^ | 0.2 ± 0.0 ^a^ | 0.2 ± 0.0 ^a^ | 0.3 ± 0.0 ^a^ | 0.1 ± 0.0 ^a^ | 10.1 ± 0.7^b^ |
| **C20:0** | 0.3 ± 0.3 ^a^ | nd | 0.6 ± 0.2 ^a^ | 0.9 ± 0.1 ^b^ | 0.2 ± 0.0 ^a^ | 1.8 ± 0.3 ^c^ | 0.4 ± 0.1 ^a^ | 0.7 ± 0.0 ^a^ | 1.1 ± 0.1 ^b^ | 0.2 ± 0.0 ^a^ |

nd = not determined
